# Association between SYNTAX Score and Coronary Collateral Circulation

**DOI:** 10.5935/abc.20170156

**Published:** 2017-11

**Authors:** Levent Cerit

**Affiliations:** Near East University - Nicosia - Cyprus

**Keywords:** Myocardial Infarction, Coronary Circulation / physiopathology, Coronary Angiography, Coronary Artery Disease, Probability

**Dear Editor,**

I have read the article entitled “Which Coronary Lesions Are More Prone to Cause Acute Myocardial Infarction?” by Sen et al. with great interest, recently published in journal. The investigators reported that more than 70% of the patients with acute myocardial infarction (MI) had coronary collateral circulation (CCC) with Rentrop scores of 1-3 during primary coronary angiography. This shows that most cases of acute MI in our study originated from underlying high-grade stenosis, challenging the common believe. Higher serum triglycerides levels, greater mean platelet volume, and increased white blood cell and neutrophil counts were independently associated with impaired development of collateral vessels.^1^

Synergy between percutaneous coronary intervention with Taxus and cardiac surgery (SYNTAX) score is the angiographic scoring system and is widely used to evaluate the severity and complexity of coronary aretry disease.^2^ SYNTAX score (SS) predicts not only possible peri-procedural difficulties but also indicates the pattern of atheroma including length, thrombosis, and calcification of the lesion.^3^ Association between multi-vessel disease and CCC has been reported by several studies.^4,5^ Börekçi et al.^4^ reported that higher SS in patients with poor CCC. Cetin et al.^5^ observed that in the poor CCC group, SS were significantly higher compared to good CCC group.

In this context, considering association between SS and CCC, correlation of this study’s result with SS may shed light on further studies.
