# Supplementary material for: Association between SYNTAX Score and Coronary Collateral Circulation
Source: Arq Bras Cardiol. 2017 Nov;109(5):497–8. doi: 10.5935/abc.20170156 (PMC5729789; doi:10.5935/abc.20170156)
Supplement: Related texts

# Reply

**Authors:** Taner Sen

**Dear editor**

I have read the letter to the editor about my study entitled “Which Coronary Lesions Are More Prone to Cause Acute Myocardial Infarction?” with great interest. The author proposed that high Syntax score might be correlated with poor coronary collateral development.

Coronary collateral vessels serve as conduit between proximal site of occluded coronary artery and its distal parts.^1^ The most important triggered effect of the collateral vessel development is the shear stress due to occlusion. 20-25% of normal humans have coronary collateral circulation but it is weak and small and it cannot be seen during coronary angiography. Pressure gradient after occlusion or severe stenosis of a coronary artery lead to endotelial stimulation and arteriogenesis and also enlargement of preexisting collateral vessels.^2^

In the study performed by Borekci et al.^3^ they found that patients with poorly developed collateral coronary circulation had higher SYNTAX scores compared with the well-developed coronary collateral group. However, multivariate analysis revealed that there was no relationship between SYNTAX score and coronary collateral flow. So far, there has not been a specific study addressing this issue. In our study, we did not calculate the SYNTAX score because it was not the aim of the study. I think that other specific trials addressing this issue are needed to determine if there is any relationship between the SYNTAX score and the coronary circulation or not. In addition, the possible mechanism of this relationship has to be explained.

**Taner Sen**

## References

[1] Pitt B (1959). Interarterial coronary anastomoses: occurrence in normal
hearts and in certain pathologic conditions. Circulation.

[2] Seiler C (2010). The human coronary collateral circulation. Invest.

[3] Börekçi A, Gür M, Şeker T, Baykan A, Özaltun B, Karakoyun S (2015). Coronary collateral circulation in patients with chronic
coronary total occlusion; its relationship with cardiac risk markers and
SYNTAX score. Perfusion.
